# Effectiveness of the baby-friendly community initiative in promoting exclusive breastfeeding among HIV negative and positive mothers: a randomized controlled trial in Koibatek Sub-County, Baringo, Kenya

**DOI:** 10.1186/s13006-020-00299-4

**Published:** 2020-07-14

**Authors:** Betty Mogesi Samburu, Sera Lewise Young, Frederick Murunga Wekesah, Milkah Njeri Wanjohi, Judith Kimiywe, Peter Muriuki, Paula L. Griffiths, Stephen T. McGarvey, Nyovani Janet Madise, Elizabeth W. Kimani-Murage

**Affiliations:** 1 Formerly Division of Nutrition and Dietetics, Ministry of Health in Kenya, Nairobi, Kenya; 2 United Nations Children’s Fund (UNICEF) Kenya Country Office, Nairobi, Kenya; 3 Institute of Policy Research, Northwestern University, Evanston, USA; 4 Maternal and Child Wellbeing Unit, African Population and Health Research Center, Nairobi, Kenya; 5 Julius Global Health, Julius Center for Health Sciences and Primary Care, University Medical Center Utrecht, Utrecht University, Utrecht, the Netherlands; 6 Department of Foods, Nutrition and Dietetics, Kenyatta University, Nairobi, Kenya; 7 Institute of Global Health Equity Education, University of Global Health Equity, Kigali, Rwanda; 8 School of Sport, Exercise and Health Sciences, Loughborough University, Loughborough, UK; 9 International Health Institute, Brown University School of Public Health, Providence, USA; 10 African Institute for Development Policy, Lilongwe, Malawi; 11 Wellcome Trust, London, UK; 12 Human Nutrition, School of Medicine, Dentistry and Nursing, College of Medical, Veterinary & Life Sciences, University of Glasgow, Glasgow, G31 2ER UK; 13 Stellenbosch Institute for Advanced Study (STIAS), Wallenberg Research Centre, Stellenbosch University, Stellenbosch, South Africa

**Keywords:** Baby friendly community initiative, Exclusive breastfeeding, HIV, Attitudes, Practices, Kenya, Peer counsellors

## Abstract

**Background:**

Although the baby-friendly community initiative (BFCI) has been proposed as a community-level approach to improve infant feeding practices, there is little data on its variation in effectiveness by HIV status. We conducted a study to determine the effectiveness of BFCI in changing knowledge and attitudes towards exclusive breastfeeding (EBF) and increasing the rates among HIV negative and HIV positive women in rural Kenya.

**Methods:**

A community-based cluster-randomized controlled trial was implemented from April 2015 to December 2016 among 901 women enrolled across 13 clusters. The intervention groups received a minimum of 12 personalized home-based counselling sessions on infant feeding by trained community health volunteers from their first or second trimester of pregnancy until 6 months postpartum. Other interventions included education sessions at maternal child clinics, mother-to-mother support group meetings and bi-monthly baby-friendly gatherings targeting influencers. The control group received standard health education at the facility and during monthly routine home visits by community health volunteers not trained on BFCI. Primary outcome measures were the rates of EBF at week 1, months 2, 4 and 6 postpartum. Secondary outcomes included knowledge and attitudes regarding breastfeeding for HIV-exposed infants. Statistical methods included analysis of covariance and logistic regression.

**Results:**

At 6 months, EBF rates among HIV negative mothers were significantly higher in the BFCI intervention arm compared to the control arm (81.7% versus 42.2% *p* = 0.001). HIV positive mothers in the intervention arm had higher EBF rates at 6 months than the control but the difference was not statistically significant (81.8% versus 58.4%; *p* = 0.504). In HIV negative group, there was greater knowledge regarding EBF for HIV-exposed infants in the intervention arm than in the control (92.1% versus 60.7% *p* = 0.001). Among HIV positive mothers, such knowledge was high among both the intervention and control groups (96% versus 100%, *p* > 0.1). HIV negative and positive mothers in the intervention arm had more favourable attitudes regarding EBF for HIV-exposed infants than the control (84.5% versus 62.1%, *p* = 0.001) and (94.6% versus 53.8% to *p* = 0.001) respectively.

**Conclusions:**

BFCI interventions can complement facility-based interventions to improve exclusive and continued breastfeeding knowledge, attitudes, and behaviours among HIV negative and positive women.

## Background

Exclusive breastfeeding (EBF) has been identified as the single most effective intervention for child survival, with the potential to prevent 13% of under-five deaths annually in low- and middle-income settings [1, 2]. The World Health Organization (WHO) and UNICEF recommend optimal infant and young child feeding as immediate and early initiation of breastfeeding within the first hour of birth, EBF for the first 6 months of life, and continued breastfeeding from 6 months to 2 years or beyond alongside nutritionally adequate, appropriate and safe complementary foods [3]. This recommendation applies to both HIV-unexposed and –exposed infants in low- and middle-income countries (LMICs) [4, 5].

Early initiation in the first hour of life and within 1 day has been shown to avert 22 and 16% of all neonatal deaths respectively [2]. Children who are not breastfed exclusively have a five- and seven-fold increased risk of death due to pneumonia and diarrhoea respectively [4, 6, 7]. Not breastfeeding contributes to 72% of all admissions for diarrhoea and 57% for respiratory infections [8]. Long-term health benefits of breastfeeding include reduced risk of obesity, allergies, heart disease and diabetes into adulthood [9, 10].

Despite the high quality evidence of the benefits of breastfeeding, globally only 42 and 41% of children are initiated to the breast within the first hour after birth and exclusively breastfed respectively in the first 6 months [2, 4, 11]. In LMICs, 50% of children are initiated to the breast within 1 h of birth and only 36% are exclusively breastfed for 6 months [2].

In Kenya, the proportion of children younger than 6 months who are exclusively breastfed has markedly increased over the years. Early initiation of breastfeeding increased from 58 to 62% while EBF increased from 32% in 2008 to 61% in 2014 [12, 13].

The increment could have been attributed to strong policy roadmaps where a number of strategic documents and legislations such as the maternal infant and young child nutrition (MIYCN) strategy 2012–2017, the 2012 breast milk substitute regulation and control Act, and the workplace support for breastfeeding legislation and guidelines which have been developed and implemented through government and donor support in addition to robust monitoring system for MIYCN [14, 15]. Further, there has been an increase in health facility deliveries following the free maternity policy in 2013 and this could have had an impact, particularly on early initiation of breastfeeding [16]. However, regional disparities in breastfeeding practices remain [13].

Breastfeeding rates are still low among HIV-infected mothers. Approximately 59% of HIV-exposed infants less than 6 months old are exclusively breastfed, and only 32% are breastfed up to 1 year [17]. In Kenya, the recommendations for HIV-exposed infants were to breastfeed to just 1 year [17, 18], but in 2018 this was revised to 2 years of age [5].

Among the infant feeding challenges faced by HIV-infected mothers is fear of HIV transmission [19] which can lead to sub-optimal infant feeding [20, 21]. Fear may lead health care providers to counsel mothers based on their own personal beliefs, discouraging EBF, often leading mothers to make feeding decisions based on fear of HIV transmission, instead of the promotion of child survival [22]. Fear may act as a barrier to EBF adherence where mothers opt to mixed-feeding as a cultural norm.

Since the introduction and widespread use of antiretroviral (ARVs) drugs, the risk of mother-to-child transmission (MTCT) rates of HIV drastically reduced to < 2%. The PROMISE trial in South Africa found the transmission risk when the mother was on combination antiretroviral therapy (ART) to be as low as 0.3% at 6 months, and 0.7% at 12 months [23]. A systematic review and meta-analysis conducted on postnatal HIV transmission in breastfed infants of HIV infected women on ART found significant reduced postnatal transmission rates of 1.08 (95% CI: 0.32, 1.85) at 6 months and 2.93 (95% CI: 0.68, 5.18) at 12 months [24].

Despite the evidence of reduced risk of transmission, HIV positive breastfeeding mothers face many challenges including those that may compromise their adherence to ARVs. These include stigma [25–27], failure of disclosure of HIV status [28], and family and peer pressure [29, 30]. These challenges become barriers to breastfeeding for HIV positive mothers in the community, even though EBF is the common choice for them as they receive counselling at the health facility. HIV positive breastfeeding women therefore require additional support from health services and from the community [4]. Without this additional support, it is likely that women will practice mixed feeding contrary to the guidelines on infant feeding in the context of HIV as well as non-adherence to ARV drugs.

The baby friendly hospital initiative (BFHI) has been recommended by WHO and UNICEF as one of the strategies to be used to promote, protect and support breastfeeding. The BFHI recommends integration of HIV services and extension to the community through the tenth step [31, 32]. The initiative has positive impacts on early initiation and early EBF especially in a hospital setting [33]. However, the impact of BFHI may be limited in developing country settings like Kenya where 39 and 60% of women in urban and rural areas respectively, deliver at home [12, 13].

Recognizing the need to reach women who don’t deliver at the hospital, as well as the need to foster support among the broader community, the Ministry of Health (MOH) in Kenya looked to the baby friendly community initiative (BFCI). The Ministry developed BFCI implementation guidelines which by 2019 were being implemented in 33 out of 47 counties [34]. An example of BFCI implementation in Kenya is documented for Migori and Kisumu counties in their routine data collection [34, 35]. The guidelines contain an 8-point plan used as a guide to educate and counsel mothers on infant feeding:

1. Have a written MIYCN policy summary statement that is routinely communicated to all health care providers, community health volunteers and the community.
2. Train all health-care providers and community health volunteers in the knowledge and skills necessary to implement the MIYCN policy.
3. Promote optimal maternal nutrition among women and their families.
4. Inform all pregnant women and their families about the benefits of breastfeeding and risks of artificial feeding.
5. Support mothers to initiate breastfeeding within the first 1 h of birth, establish and maintain EBF for first 6 months.
6. Encourage sustained breastfeeding beyond 6 months to 2 years or more alongside timely introduction of appropriate, adequate and safe complementary foods.
7. Provide a welcoming and conducive environment for breastfeeding families.
8. Promote collaboration between healthcare staff, MIYCN support groups and the local community.

BFCI is being implemented in LMICs including Gambia and Cambodia [35–39]. Kenya also adopted the BFCI as a strategy to improve MIYCN practices at the community level and a country implementation guideline was developed [34]. However, little evidence on its effectiveness in improving breastfeeding practices exists either for the general population or specifically for HIV positive mothers. This study therefore sought to determine the effectiveness of BFCI in improving breastfeeding knowledge, attitudes and practices among HIV negative and positive mothers in rural communities. The primary hypothesis was whether the rates of EBF would be higher among HIV negative and positive mothers who received the BFCI intervention when compared to those who did not receive it. The secondary hypotheses were to test whether both correct knowledge regarding EBF for HIV-exposed infants, and more favourable attitudes regarding breastfeeding for HIV-exposed infants would be higher among HIV-infected and uninfected mothers who received the intervention compared to those who did not.

## Methods

### Study site

The study was conducted in Koibatek, one of six sub-Counties in Baringo County, Kenya. The sub-County has an approximate total population of 125,637 with 30,203 women of reproductive age (15–49) and 4799 children under 1 year. The EBF rate for under 6 months in 2015 was 37%, below the national rates of 61% [40].

The prevalence of HIV infection among pregnant women in Baringo County in 2015 was estimated to be 3% with a total of 313 mothers living with HIV [41]. The County was ranked 25 out of 47 counties in Kenya in terms of contribution to new HIV infection. In addition, it ranked 21 out of 47 counties in Kenya on HIV prevalence and having small improvement in HIV infection reduction of less than < 50% as compared to 14 counties which made progress of > 50% HIV reduction in the same year [42, 43].

### Study design

The study was part of a bigger community-based cluster randomized trial implemented between October 2014 and July 2017 to test the feasibility and effectiveness of BFCI in rural Kenya in order to inform scale up [44]. Training of healthcare providers and community health volunteers (CHVs) and baseline data collection were conducted January to March 2015, while prospective data collection was carried out from April 2015 to December 2016. Qualitative formative data were collected in August 2014 and the findings used to inform the intervention design.

Cluster randomization was used as opposed to individual randomization as this would minimize ‘contamination’ of information across the study groups. The BFCI is also inherently a community-based program including community and mother support groups meaning that the intervention needed to be allocated at the level at which it would be implemented.

Community units (CUs), established under the MOH community health strategy, were the unit of clusters. Each CU has a population of approximately 5000 and is served by 25 CHVs. There are several villages within one CU and each CHV is allocated between 100 and 150 households depending on the distance and geographical location [45]. The 13 clusters were randomly allocated into the BFCI intervention arm (*n* = 6) and control arms (*n* = 7) by a biostatistician with no information on the CUs based on a ratio of 1:1 using Microsoft Excel. The additional extra CU for the control was also allocated randomly.

### Sample size determination

We designed the study to be able to detect an 18% increase in the prevalence of EBF, based on effects observed in prior similar studies testing effectiveness of community based interventions on EBF [46]. With a two-sided 5% significance level and a power of 80%, a sample size of 780 mother and child pairs in total was required. An additional 10% at the intervention and control was considered respectively to account for loss to follow-up. A design effect of 4 based on an intra-cluster correlation of 0.05, and previous research on breastfeeding practices in some settings in Kenya and an average cluster size of 62.5 was used [44]. The target was to recruit 400 mothers in each of the study arms. Nine Hundred and one (901) women were ultimately recruited out of the targeted 800. Twelve CUs were required for the estimated sample size. The 13th CU was randomly assigned to the control arm.

### Study eligibility

Any pregnant woman (15–49 years) in their first or second trimester residing in the 13 CUs who intended to stay in the CUs for 6 months or more after delivery was potentially eligible to take part in the study. Potential participants were identified by the CHVs at the community or through the facility by the community health extension worker (CHEWs). They were then referred to the data collector for eligibility screening, consent, and baseline interview.

### Blinding

The investigator and the CHVs were aware of the interventions given to the experimental groups. The hypothesis of the study could not be concealed from the CHVs since they were offering education and counselling to mothers. Field workers knew about control and intervention groups but were independent of the intervention delivery. The mothers were not told of the different treatment groups.

### Training and mentorship of study staff

A five-day BFCI training was conducted by six master trainers who were MOH employees and accredited trainer of trainers (TOTs). A total of 25 participants composed of the sub-County health management team (SCHMT), health workers from the sub-County, and two CHEWs from each of the 6 intervention units were trained**.** The training content was based on the BFCI implementation package for Kenya that had been developed by the MOH, adopted from the WHO/UNICEF integrated infant and young child feeding counselling course [34, 47]. The key messages for counselling were covered in each of the 8 steps for BFCI, including infant feeding in the context of HIV [48]. Other key messages in the training that were revealed to be important through formative work for this study and elsewhere [21] included male involvement, and the common tradition of giving prelacteal feeds.

The CHVs were trained by the CHEWS under the supervision of master trainers. The CHVs are volunteers providing community health services as established under the community health initiatives [49]. They conduct home visits and other community-based activities e.g. community dialogues, action days and also collect community-based data for health information systems. A total of 25 CHVs from each of the 6 intervention CUs underwent the training conducted at the community level either in a nearby church or a school.

The CHVs were also trained on their roles and responsibilities in the study including counselling skills, establishment and conducting of monthly mother-to- mother support group (MTMSG) meetings and nutrition education to mothers. Thirteen field research assistants and one supervisor also underwent a seven-day training including field experiences on data collection. The questionnaires were subjected to multiple pre-testing during their development and finally pilot tested and refined for clarity and accuracy.

Upon completion of the training, the SCHMT, CHEWS and CHVs developed a joint work plan for rollout of the BFCI. The rollout included: formation and facilitation of community mother support groups (CMSGs) targeting pregnant and lactating mothers including influencers, provision of counselling and information and education materials, development of a framework for mentorship and supportive supervision, promotion, protection and support of breastfeeding.

The paper presents findings from a community-based intervention in which trained CHVs were used to offer community-based intervention on MIYCN to pregnant mothers from their first or second trimester to 6 months post-delivery. We describe the outcomes in terms of EBF rates for those in the intervention and control groups, stratified by HIV status of the mother, which was ascertained after mothers were recruited to either the intervention or the control groups. We also report on knowledge and attitudes regarding EBF for HIV-exposed infants. The primary goal of the study was to generate evidence that would be used to change breastfeeding practices for HIV negative and HIV positive mothers, and improve knowledge for community support towards elimination of mother to child transmission (eMTCT) in addition to informing policy. 

### Activities in the BFCI intervention group

The mothers in the BFCI intervention group received a minimum of 12 home-based counselling sessions by trained CHVs. The CHV visited the pregnant woman after she had enrolled and completed the baseline survey. Visits occurred approximately once every month up to week 37 of pregnancy, and thereafter every 2 weeks through the first month postpartum, then once a month until the infant was 6 months old. The times for visits were scheduled between the CHVs and the mother at her convenience.

At these visits, the CHVs used counselling cards with the content structured around the eight-step point plan for BFCI which included good maternal nutrition and early antenatal care (ANC), importance of EBF including early initiation, feeding of colostrum, attachment and positioning, prevention of mother to child transmission (PMTCT), solving breastfeeding difficulties and family support. The CHVs also had a counselling checklist to ensure all areas were covered depending on the stage of counselling.

Other activities included weekly scheduled education sessions on infant and young child feeding at the maternal child health clinic which targeted mothers who attended the clinic that particular day within a CU catchment area, monthly MTMSG meetings, and bi-monthly baby friendly community gatherings targeting all influencers which were conducted by established community mother support groups members. Breastfeeding corners/rooms were also established at the primary health care facility where mothers would meet and share experiences when they came for ANC clinic. Simplified information, pictures, illustrations and messages translating the policy into local languages were placed in the breastfeeding corners and mothers were mostly taught by CHEWs or CHVs in these corners.

Ongoing mentorship and supportive supervision by the SCHMT to the CHEWS and CHVs took place every month in the first 6 months of study commencement and thereafter quarterly. Based on outcome of the mentorship and supportive supervision including gaps identified, actions were agreed upon by both the mentee and the mentor and review of the implementation followed up in the subsequent visits.

### Control group

The control group received standard health education at the facility. Topics on MIYCN were part of other topics given within the schedule. As such, a woman could entirely miss receiving any breastfeeding information during her ANC clinic visits if during her attendance such topics were not in the schedule for that day. They also received standard counselling delivered by CHVs not trained on MIYCN at home during the CHV routine monthly visits. The routine visits were conducted once a month for the pregnant or lactating mother. Routine data were also collected during the visits for reporting purposes. Standard care did not differ by HIV status. However, mothers diagnosed with HIV would usually receive individualized counselling on infant feeding at the PMTCT clinic.

### Data collection

The HIV status of all mothers enrolled in the study was collected from the facility records. Each individual mother enrolled was allocated a unique number during baseline recruitment by the field worker. The same number was presented to the facility during the ANC visit and recorded against each mothers ANC profile register. The HIV status could be determined from the same records for ANC profile register as it is a policy that all mothers should be tested. The data from the ANC profile were collected and linked to the same data collected in the community during home visits by data collectors.

The primary outcome was the EBF rates over the 6-month postpartum period. Breastfeeding behaviour was determined using 24-h recall interviews at each of these time points. The secondary outcome was maternal knowledge and attitude regarding breastfeeding for HIV-exposed infants which was assessed at baseline and the final data collection point at 6 months. The knowledge and attitude questionnaire was formulated from the current policy guidelines for maternal infant and young child feeding in the context of HIV for Kenya [17, 18, 50].

Knowledge was determined through answers about the recommended period of EBF, duration of continued breastfeeding and practices about breastfeeding for HIV-exposed infants. Attitudes towards breastfeeding were determined through responses to a Likert scale questionnaire with five points ranging from strongly agree to strongly disagree. The topics covered included length of EBF and overall duration of EBF.

### Data analysis

Data analysis was done using Stata Version 12.1 (Stata Corporation LP, College Station, TX). The characteristics of the study population, and outcomes such as infant feeding practices, EBF rates and maternal knowledge and attitudes regarding EBF for both HIV negative and positive mothers in the study were presented in frequencies and means. T-test was used to determine differences between intervention and control groups for continuous variables, while chi-square tests and cluster-adjusted relative risks were used to test relationships between categorical variables with the main outcomes.

Comparison of outcomes between BFCI intervention group and the control group by HIV status was done using multivariate logistic regression and multinomial logistic models that account for clustering. Statistical significance was set at *p* < 0.05.

### Ethical considerations

**Fig. 1:**
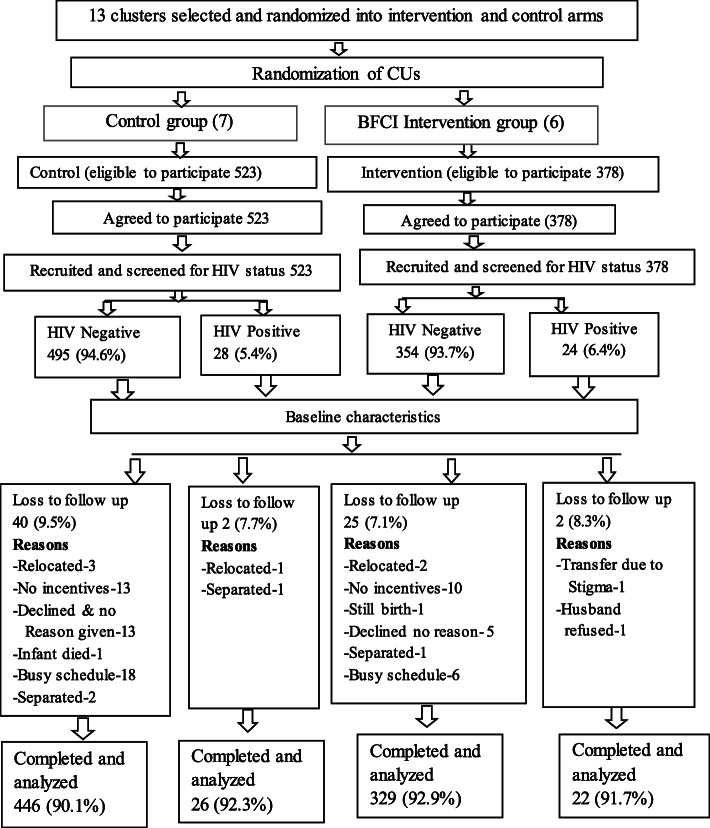
Randomization of study participants to intervention and control groups

A study permit was obtained from the National Commission for Science, Technology, and Innovations. Ethical clearance for PhD research was obtained from Kenyatta University Ethical Committee (Ref KU/R/COMM/51/678), while ethics approval for the main study was obtained from the Kenya Medical Research Institute (KEMRI) SERU (Non-SSC Protocol No. 443). In addition, approval was given by Baringo County Government to carry out the study in Koibatek sub-County. Informed consent was sought from the participants before involving them in the study. An outline of randomization of participants into intervention and control is given in Fig. 1.

## Results

A total of 901 pregnant mothers in 13 CUs were screened and enrolled in the study for the period April 2015 to December 2016 (Fig. 1). The figure gives graphical representation of the study.

An outline of demographic and socioeconomic characteristics of study participants is given in Table 1.

**Table 1:** Sociodemographic characteristics of study participants stratified by HIV status and treatment allocation

	HIV Negative				HIV Positive			
	Control ***n*** = 495	Intervention ***n*** = 354	Total ***n*** = 849	X^**2**^ ***P***-value	Control ***n*** = 28	Intervention ***n*** = 24	Total ***n*** = 52	X^**2**^ ***P***-value
**Age (mean±)**	24.4 ± 0.6	23.5 ± 0.5	24.5 ± 0.4		22.4 ± 0.4	22.5 ± 0.5	22.9 ± 0.4	
**Age**								
< 20	109(22.0)	53(15.0)	162(19.1)	0.06	6(22.7)	4(13.6)	10(19.2)	0.90
21–30	290(58.6)	220(62.0)	510(60.1)		18(63.7)	16(68.2)	34(65.4)	
> 31	96(19.4)	81(22.9)	177(20.8)		4(13.6)	4(18.2)	8(15.4)	
**Education**								
Less than Primary	79(16.0)	54(15.2)	133(15.6)	0.07	6(23.1)	3(13.6)	9(17.3)	0.80
Primary	241(48.7)	115(32.5)	356(41.9)		8(26.9)	9(36.4)	17(32.7)	
Secondary	124(25.0)	130(36.9)	254(30.0)		11(38.5)	10(40.9)	21(40.4)	
College/University	51(10.3)	55(15.4)	106(12.5)		3(11.5)	2(9.1)	5(9.6)	
**Parity**								
0	49(9.9)	35(9.8)	84(9.9)	0.91	6(21.4)	6(25.0)	12(23.1)	0.53
1–3	262(52.9)	188(53.2)	450(53.0)		15(53.6)	14(58.3)	29(55.8)	
4–6	156(31.5)	111(31.4)	267(31.4)		4(14.3)	4(16.7)	8(15.4)	
7+	28(5.6)	20(5.6)	48(5.7)		3(10.7)	0.(0.00)	3(5.8)	
**Source of livelihood**								
Own business	66(13.3)	49(13.8)	115(13.5)	0.06	3(11.5)	5(22.7)	8(15.3)	0.63
From spouse	188(37.9)	140(39.5)	328(38.6)		10(34.6)	11(45.5)	21(41.1)	
HH head	63(12.7)	45(12.7)	108(12.7)		6(19.2)	2(9.1)	8(15.3)	
Farming	90(18.2)	61(17.2)	151(17.9)		4(15.4)	4(13.6)	8(15.3)	
Formal	29(5.8)	22(6.2)	51(6.0)		1(3.9)	0(0.0)	1(1.8)	
Casual	59(11.9)	37(10.6)	96(11.3)		4(15.4)	2(9.1)	6(11.2)	
**Marital status**								
Separated/Divorced/Widowed	119(24.0)	66(18.6)	185(21.7)	0.06	5(19.2)	3(13.6)	8(15.4)	0.61
Married	376 (76.0)	288(81.4)	664(78.3)		23(80.8)	21(86.4)	44(84.6)	

### Baseline comparisons

There were no statistically significant differences observed between the BFCI intervention and control groups in terms of age, education, parity, source of livelihood and marital status (Table 1). Further, the prevalence of HIV between the intervention (6.3%) and the control groups (5.4%) was not statistically different.

### Impact of BFCI intervention on EBF rates among HIV negative and positive mothers

**Fig. 2:**
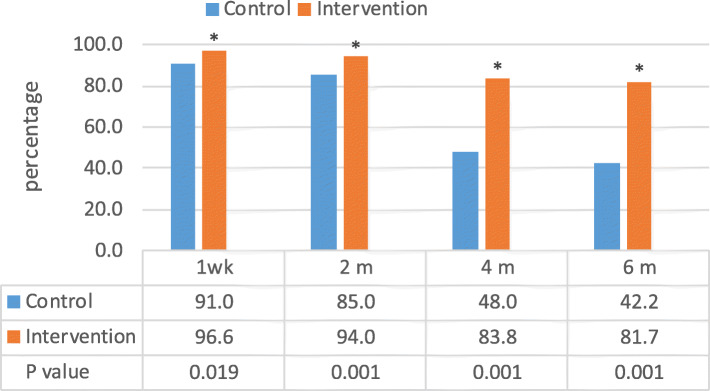
Exclusive breastfeeding rates by intervention period for HIV negative mothers

**Fig. 3:**
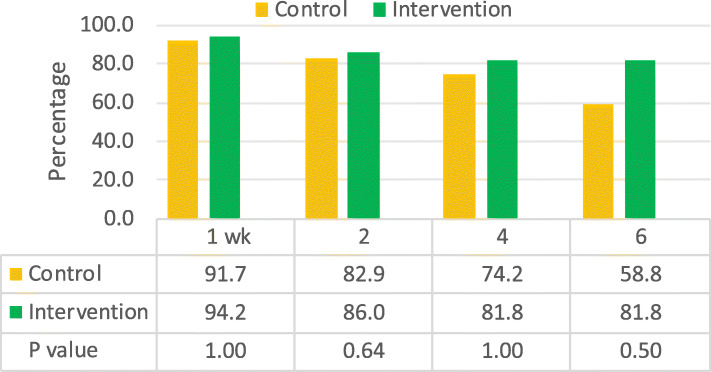
Exclusive breastfeeding rates by intervention period for HIV positive mothers

In both the intervention and control groups and for both HIV negative and HIV positive mothers, EBF rates were over 90.0% at 1 week postpartum. For HIV negative mothers, at 4 months, EBF was much lower among the control group (48.0%) compared to the intervention group 83.8% (Fig. 2). At the same time point, EBF rates among HIV positive women in the control group was lower (74.2%) compared to (81.8%) among those in the intervention (Fig. 3).

**Fig. 4:**
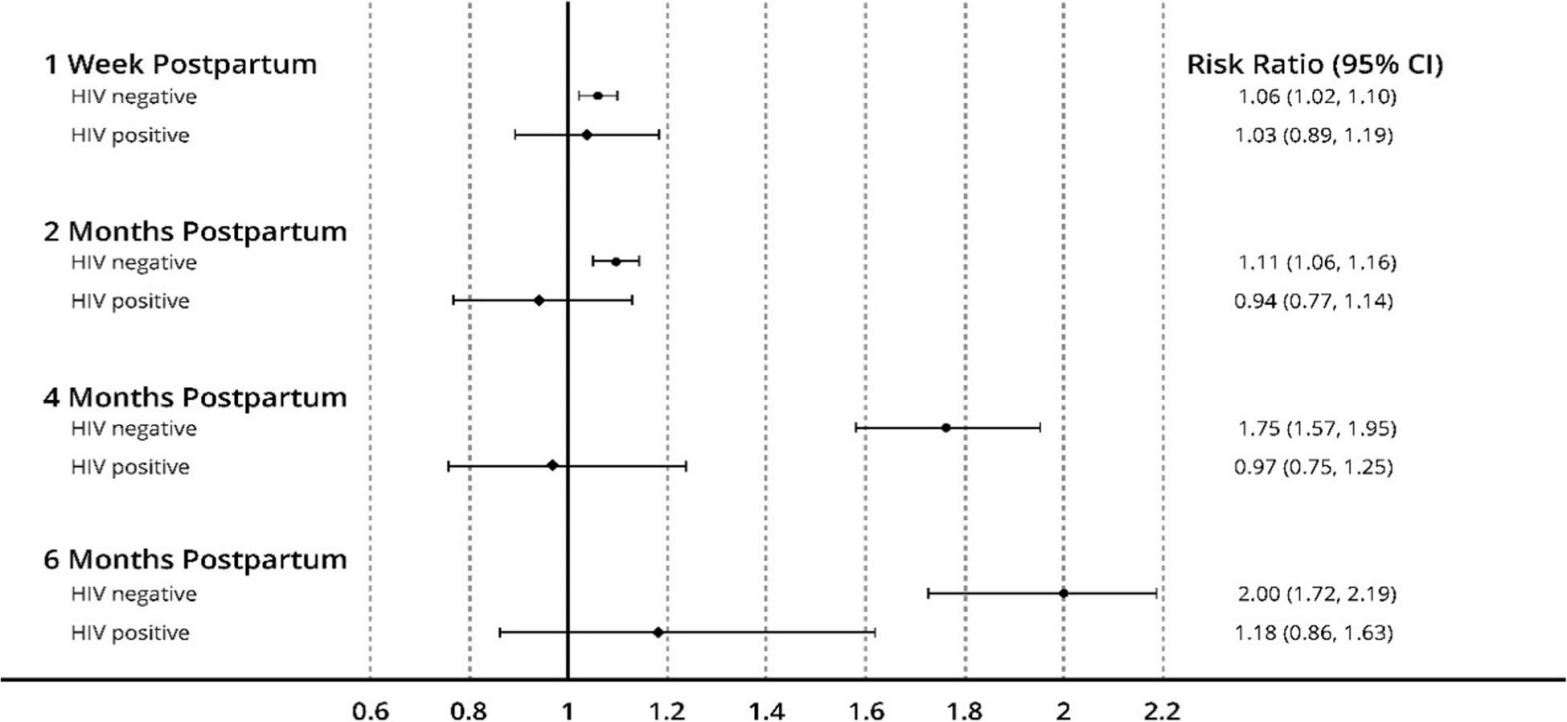
Relative risk of exclusive breastfeeding for mothers in the intervention arm of the baby-friendly community initiative in Koibatek, Kenya, by HIV status

In the HIV negative group, the prevalence of EBF was consistently statistically significantly higher among women in the intervention arm than in the control arm. For example, at 1 week, the relative risk (RR) of EBF among HIV negative women in the intervention relative to those in the control group was 1.06, (95% CI 1.02, 1.10, *p* = 0.019); at 2 months RR 1.11 (95% CI 1.06, 1.16, p= 0.001); at 4 months RR 1.75, (95% CI 1.57,1.95), p = 0.001); at 6 months, mothers in the intervention group had a two-fold increased likelihood of practicing EBF compared to those in the control group RR 2.0 (95% CI 1.40, 3.20, p= 0.001) (Fig. 4).

For the HIV positive group, the relative risk of EBF was similar between the intervention and control groups at 1 week, 2 months, and 6 months postpartum (Fig. 4).

### Impact of BFCI intervention on knowledge, attitude and EBF rates among HIV negative and positive mothers

**Fig. 5:**
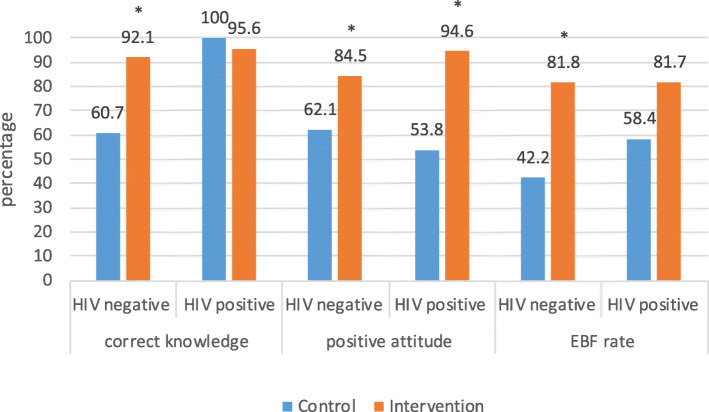
Breastfeeding knowledge, attitude and practices at 6 months by HIV status and study group

In the HIV negative group, there was significantly greater knowledge about EBF for HIV-exposed infants in the BFCI intervention arm than in the control (92.1% versus 60.7% *p* = 0.001, Fig. 5). Among HIV positive mothers, such knowledge was high among both the intervention and control groups (96% versus 100%, *p* = 0.93). Attitudes among HIV negative mothers regarding EBF for HIV-exposed infants were more positive in the intervention arm than the control (84.5% versus 62.1%, *p* = 0.001). The same pattern was observed among HIV-positive women (94.6% versus 53.8% to *p* = 0.001).

**Fig. 6:**
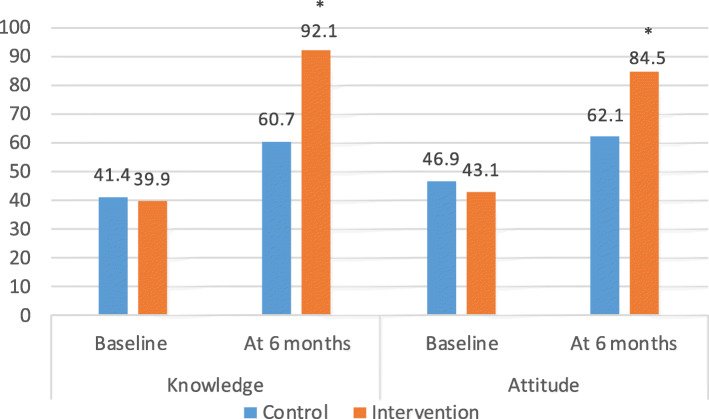
Knowledge and attitude at baseline and at 6-months post-partum for HIV negative mothers. Footnote: HIV negative mothers in the control (*n* = 495) and in the intervention (*n* = 354) at baseline. HIV negative in the control (*n* = 446) and in the intervention (*n* = 329) at 6 months

**Fig. 7:**
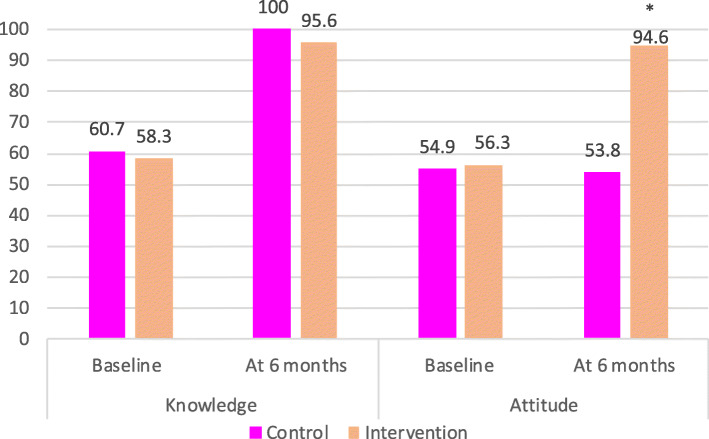
Knowledge, attitude at baseline and 6-months post-partum for HIV positive mothers. Footnote: HIV positive mothers in the control (*n* = 28) and in the intervention (*n* = 24) at baseline. HIV positive mothers in the control (*n* = 24) and in the intervention (*n* = 22) at 6 months. Chi-square test: *p* = 0.05 at 95% Cl; * *p* = 0.001

Knowledge of EBF for HIV-exposed infants was similar between the control and intervention at baseline in the HIV negative group (41.4% vs 39.9%); there was a difference of change in knowledge from 39.9% at baseline to 92.1% in the intervention group (Fig. 6). For HIV positive mothers, all in the control (100%) had knowledge on EBF. Change in attitude was greater among intervention HIV positive mothers (56.3 to 94.6%) from baseline to 6 months post-partum compared with the control group (Fig. 7).

## Discussion

The BFCI intervention, which works through personalized home-based counselling by trained CHVs, significantly improved EBF rates for HIV negative mothers at 1 week, 2, 4 and 6 months compared to the control group. Knowledge and attitude significantly improved too among HIV negative mothers in the intervention. Attitude among HIV positive mothers significantly improved.

In terms of the effects of the intervention by HIV status, there was a higher prevalence of EBF among HIV positive women than HIV negative women at 4 and 6 months postpartum. The fact that HIV positive women in both study arms received individualized infant feeding counselling during ANC visits at the PMTCT clinics, with a particular emphasis on EBF might explain the higher rates of EBF at 4 and 6 months in this group when compared to the HIV negative mothers.

Among HIV positive mothers, the prevalence of EBF declined with the child’s age in both arms, but it remained higher, although not statistically significant, in both the intervention and control group at 6 months (81.8% vs 58.8%, *p*  = 0.505, Fig. 2). This lack of statistical significance is undoubtedly due to the small sample size of HIV positive mothers (*n* = 48) which limited our power to detect differences between the intervention (*n* = 26) and control groups (*n* = 22). However, our study was not designed to test differences in the impact by HIV status. There is a need for further studies on the effectiveness of the BFCI in a larger sample of HIV positive mothers to confirm the significance of this effect.

The decline in EBF rates especially from 4 months to 6 months postnatally (48.0 to 42.2%) and (74.2 to 58.4%) for HIV negative and positive mothers respectively suggests that more support is needed for those receiving usual care (in our control groups) whether HIV negative or positive.

Rates of EBF decreased with the age of the baby, but less so in the intervention, suggesting that the BFCI is a powerful tool necessary for achieving infant and young child feeding goals. This study has demonstrated that BFCI is an effective intervention strategy at the community level that may impact the improvement of EBF rates in the general population. HIV positive mothers may also benefit from the intervention although further testing is required in a larger sample of HIV positive mothers to confirm the evidence. The findings also confirm the need for health services support to HIV positive mothers to enable them to exclusively breastfeed as given in the new WHO guidance [4].

The findings of the present trial in Kenya are in agreement with those of other studies which have consistently demonstrated the effectiveness of home-based counselling and MTMSG in increasing the prevalence of EBF [35, 51–53]. In Maternal and Child Survival Program (MCSP) and UNICEF areas implementing BFCI in Migori Kenya, BFCI interventions improved EBF rates from 85.9 to 89.3% [35]. This was observed from routine health data from the programme. However, the data only captured those children attending the MCH clinics and was not population based hence it is difficult to attribute the results to the programme [53]. Another study conducted in Kenya found mothers in the MTMSG were twice as likely to breastfeed exclusively at 6 months compared to mothers in the control group, RR = 2.42 (95% CI 1.36, 4.28; *p* = 0.004) and RR = 1.89 (95% CI 1.02, 3.49; *p* = 0.033) respectively [53]. Significantly improved EBF rates were also found in an intervention study conducted in Burkina Faso, Uganda, and South Africa where in the intervention group mothers received one antenatal breastfeeding peer counselling visit and four post-delivery visits by trained peer counsellors [51]. The prevalence of EBF at 6 months based on 24-h recall were 286 (73%) in the intervention cluster and 88 (22%) in the control cluster in Burkina Faso RR = 3.33 (95%CI 1.74, 6.38); 232 (59%) and 57 (15%), respectively, in Uganda RR =3.83 (95% CI 2.97, 4.95); and 12 (2%) and two (< 1%), respectively, in South Africa RR =5.70 (95% CI 1.33, 24.26). Similar findings were noted in a systematic review and meta-analysis on interventions to improve breastfeeding outcomes [1, 54].

The findings of this study can have significant implication for national nutrition initiatives in the promotion of optimal infant feeding practices and thus child survival. However, there are some limitations in the study particularly around the small sample size which stop us from drawing definite conclusions on the impact of BFCI on improving EBF practices among HIV infected mothers.

## Conclusions

The findings of this study reveal that both HIV positive and negative mothers need continued support for sustained EBF beyond the facility level. While intensive counselling at health facility level has been given to HIV positive mothers, this study reveals that when they go to the community, they encounter similar challenges to mothers who do not visit health facilities which hinder them from exclusively breastfeeding despite having the relevant knowledge. It is evident that mothers require continued support for sustained breastfeeding beyond their discharge from hospital.

The barriers to infant feeding operate at different levels of the individual, interpersonal, community, home and health care setting. Hence a combination of strategies would be appropriate to address these barriers at these different levels. The BFCI, which used a combination of interventions including home visits, mother-to-mother support group meetings, community gatherings and campaigns, holds great promise to complement facility-based interventions such as the BFHI and elimination of mother to child transmission (eMTCT) programmes.

Optimal infant-feeding practices are the result of women’s knowledge, attitudes and behaviour, as well as the support and involvement of their partners, family members, and the community, and the BFCI appears to help align the community around the noble goal of breastfeeding. As such these findings offer support for the roll-out of BFCI to the community and strengthening of community-based promotion of breastfeeding.

## Data Availability

The data are available from the African Population and Health Research Center (APHRC) upon reasonable request and with permission of APHRC.

## Abbreviations

EBF: Exclusive breastfeeding
ARV: Antiretroviral
MTCT: Mother-to-child Transmission
BFHI: Baby friendly hospital initiative
BFCI: Baby friendly community initiative
MIYCN: Maternal infant and young child nutrition
MTMSG: Mother-to-mother support group
CU: Community unit
SCHMT: Sub-County Health Management Team
CHEW: Community health extension worker
CHV: Community health volunteer
ANC: Antenatal care
PMTCT: Prevention of Mother to Child Transmission
eMTCT: Elimination of Mother-to-Child Transmission
APHRC: African Population and Health Research Center
